# Supplementary material for: Does Awareness of Malaysian Healthy Plate Associate with Adequate Fruit and Vegetable Intake among Malaysian Adults with Non-Communicable Diseases?
Source: Nutrients. 2023 Dec 8;15(24):5043. doi: 10.3390/nu15245043 (PMC10745645; doi:10.3390/nu15245043)
Supplement: Supplementary file 1 [file nutrients-15-05043-s001.zip › nutrients-2717067-supplementary.pdf]

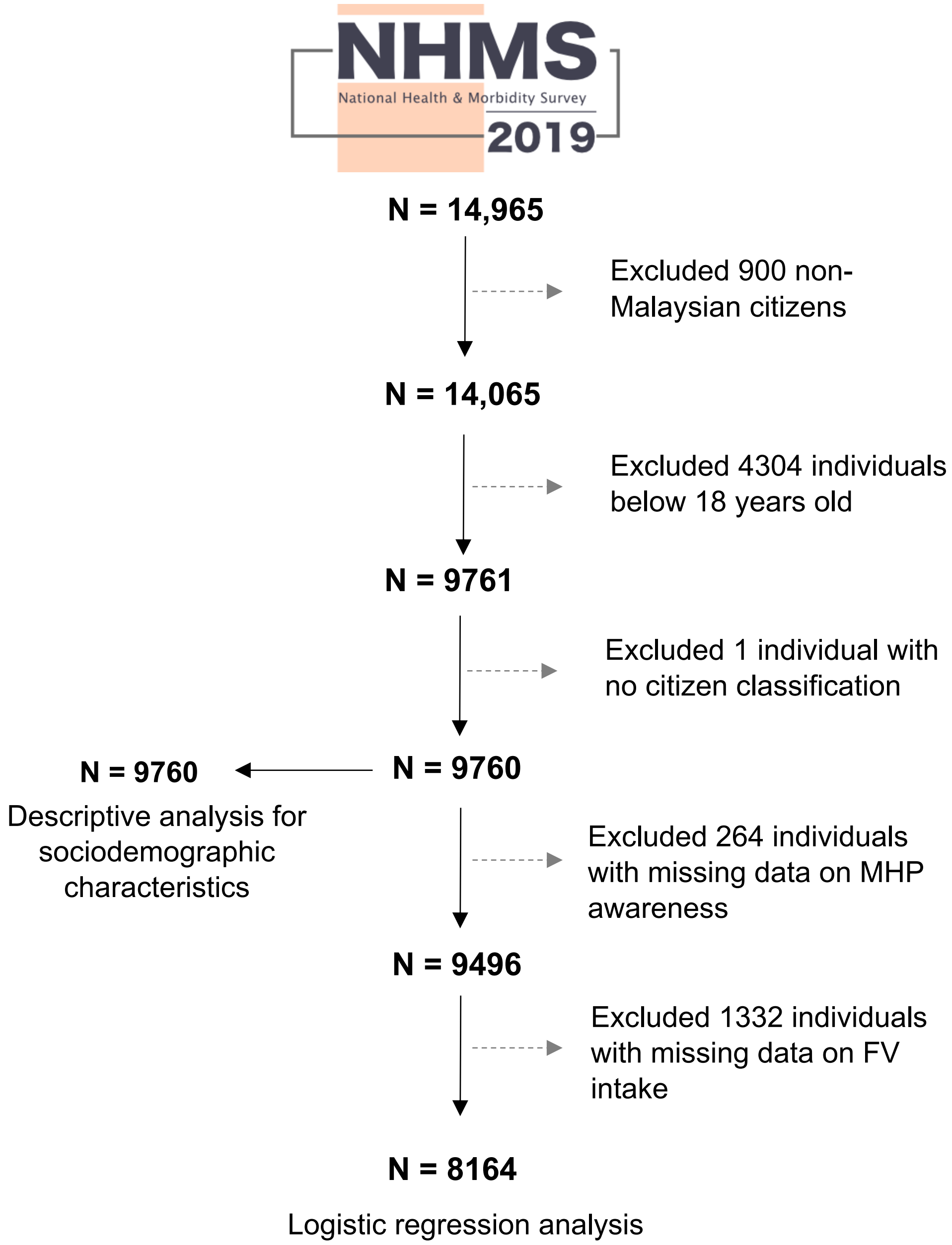

Figure S1. The inclusion and exclusion process of the NHMS respondents for data analysis.

Table S1. The prevalence of general obesity, abdominal obesity, diabetes mellitus, hypertension, and hypercholesterolemia among the Malaysian adults aged 18 years and above.

	Estimated Population	Count (n)	Prevalence (%)	95% CI[1]
General obesity	11,800,453	6487	67.1	65.3-69.0
Abdominal obesity	9,471,380	5429	50.4	48.5-52.3
Diabetes mellitus	1,929,344	1365	10.3	9.5-11.1
Hypertension	3,268,660	2327	17.4	16.2-18.6
Hypercholesterolemia	2,778,741	1960	14.8	13.8-15.8

[1]95% CI: 95% confidence interval

Table S2. The prevalence of MHP awareness among the Malaysian adults aged 18 years and above with general obesity, abdominal obesity, diabetes mellitus, hypertension, and hypercholesterolemia.

	Prevalence (%)	95% CI[1]
General obesity		
Non-obese	20.7	18.0-23.6
Obese	24.9	23.0-26.8
Abdominal obesity		
Non-obese	20.0	18.2-22.2
Obese	25.7	23.7-27.9
Diabetes mellitus		
Yes	22.9	20.0-26.2
No	22.4	20.8-24.1
Hypertension		
Yes	20.0	17.5-22.6
No	23.0	21.3-24.8
Hypercholesterolemia		
Yes	22.9	20.3-25.7
No	22.4	20.8-24.1

[1]95% CI: 95% confidence interval

Table S3. The prevalence of adequacy of FV among the Malaysian adults aged 18 years and above with general obesity, abdominal obesity, diabetes mellitus, hypertension, and hypercholesterolemia.

	Prevalence (%)	95% CI[1]
General obesity		
Non-obese	2.4	1.6-3.7
Obese	2.3	1.8-2.9
Abdominal obesity		
Non-obese	2.2	1.6-3.1
Obese	2.3	1.7-3.0
Diabetes mellitus		
Yes	2.3	1.3-4.0
No	2.4	1.9-2.9
Hypertension		
Yes	2.6	1.8-3.8
No	2.3	1.8-2.9
Hypercholesterolemia		
Yes	2.8	1.9-4.0
No	2.3	1.8-2.9

[1]95% CI: 95% confidence interval

Table S4. The association analysis between MHP awareness and adequate FV intake among the Malaysian adults aged 18 years and above.

Characteristic	aOR[1]	95% CI[1]	p-value
MHP awareness			
Not aware	1.00	—	
Aware	1.44	0.85-2.43	0.17
Gender			
Male	1.00	—	
Female	1.54	0.83-2.82	0.17
Age group (years old)			
18-39	1.00	—	

40-59	0.72	0.41-1.27	0.26
≥ 60	1.26	0.58-2.75	0.56
Ethnicity			
Malay	1.00	—	
Chinese	1.84	1.07-3.16	0.03
Indian	1.18	0.50-2.82	0.70
Others	1.01	0.47-2.15	0.99
Residential area			
Urban	1.00	—	
Rural	1.05	0.62-1.77	0.85
Marital status			
Single	1.00	—	
Married	1.30	0.72-2.36	0.38
Widow/Widower/Divorcee	1.22	0.41-3.62	0.72
Education level			
No formal education	1.00	—	
Primary education	1.18	0.25-5.62	0.84
Secondary education	1.63	0.32-8.35	0.56
Tertiary education	1.46	0.25-8.63	0.67
Occupation status			
Employed	1.00	—	
Unemployed	0.75	0.41-1.39	0.37
Monthly household income			
Bottom 40%	1.00	—	
Middle 40%	1.03	0.63-1.69	0.89
Top 20%	1.72	0.84-3.50	0.14
Smoking			
Ever smoker	1.00	—	
Never smoker	0.80	0.42-1.55	0.51

[1]aOR: adjusted odds ratio, 95% CI: 95% confidence interval

Table S5. The association analysis between MHP awareness and adequate FV intake among the Malaysian adults aged 18 years and above with general and abdominal obesity.

Characteristic	General obesity (n = 6487)			Abdominal obesity (n = 5429)		
	aOR[1]	95% CI[1]	p-value	aOR[1]	95% CI[1]	p-value
MHP awareness						
Not aware	1.00	—		1.00	—	
Aware	1.59	0.91-2.75	0.10	1.86	1.05-3.29	0.03
Gender						
Male	1.00	—		1.00	—	
Female	1.54	0.69-3.40	0.29	1.40	0.57-3.43	0.47
Age group (years old)						
18-39	1.00	—		1.00	—	
40-59	0.60	0.28-1.32	0.20	0.39	0.16-0.96	0.04
≥ 60	1.39	0.53-3.62	0.50	1.34	0.47-3.86	0.58
Ethnicity						
Malay	1.00	—		1.00	—	
Chinese	1.41	0.64-3.12	0.39	2.15	0.98-4.71	0.06
Indian	1.18	0.48-2.90	0.71	1.40	0.54-3.59	0.49
Others	1.03	0.45-2.37	0.94	1.20	0.46-3.14	0.71
Residential area						
Urban	1.00	—		1.00	—	
Rural	1.07	0.58-1.98	0.83	1.07	0.52-2.23	0.85
Marital status						
Single	1.00	—		1.00	—	
Married	1.06	0.49-2.28	0.88	0.84	0.36-1.96	0.69
Widow/Widower/Divorcee	0.88	0.26-2.94	0.83	0.65	0.18-2.32	0.50
Education level						
No formal education	1.00	—		1.00	—	
Primary education	1.01	0.14-7.46	>0.99	1.61	0.21-12.61	0.65
Secondary education	1.75	0.22-13.84	0.59	1.97	0.22-17.96	0.55
Tertiary education	1.15	0.11-11.60	0.90	1.83	0.17-20.30	0.62

Occupation status						
Employed	1.00	—		1.00	—	
Unemployed	0.56	0.30-1.02	0.06	0.47	0.24-0.92	0.03
Monthly household income						
Bottom 40%	1.00	—		1.00	—	
Middle 40%	0.96	0.51-1.80	0.89	1.34	0.70-2.59	0.38
Top 20%	1.94	0.82-4.60	0.13	1.43	0.53-3.83	0.48
Smoking						
Ever smoker	1.00	—		1.00	—	
Never smoker	0.63	0.30-1.35	0.23	1.03	0.36-2.90	0.96
Diabetes mellitus						
No	1.00	—		1.00	—	
Yes	0.98	0.49-2.00	0.97	1.01	0.47-2.16	0.99
Hypertension						
No	1.00	—		1.00	—	
Yes	1.54	0.76-3.13	0.23	2.38	1.11-5.08	0.03
Hypercholesterolemia						
No	1.00	—		1.00	—	
Yes	1.37	0.64-2.95	0.41	1.15	0.52-2.55	0.73
Abdominal obesity						
Non-obese	1.00	—				
Obese	0.98	0.53-1.78	0.94			
General obesity						
Non-obese				1.00	—	
Obese				0.89	0.37-2.15	0.80

[1]aOR: adjusted odds ratio, 95% CI: 95% confidence interval. The model predicted values for general obesity and abdominal obesity were 97.6% (AUC= 0.60 (95% CI: 0.54-0.65, p < 0.001) and 97.6% (AUC=0.64, 95% CI : 0.58-0.70, p < 0.0001), respectively.

Table S6. The association analysis between MHP awareness and adequate FV intake among the Malaysian adults aged 18 years and above with diabetes mellitus, hypertension and hypercholesterolemia.

Characteristic	Diabetes mellitus (n = 1365)			Hypertension (n = 2327)			Hypercholesterolemia (n = 1960)		
	aOR[1]	95% CI[1]	p-value	aOR[1]	95% CI[1]	p-value	aOR[1]	95% CI[1]	p-value
MHP awareness									
Not aware	1.00	—		1.00	—		1.00	—	
Aware	6.88	2.13-22.18	<0.01	4.39	1.96-9.83	<0.001	4.16	1.48-11.72	<0.01
Gender									
Male	1.00	—		1.00	—		1.00	—	
Female	0.79	0.12-5.15	0.81	0.89	0.27-2.91	0.84	0.78	0.23-2.58	0.68
Age group (years old)									
18-39	1.00	—		1.00	—		1.00	—	
40-59	0.14	0.03-0.77	0.02	0.23	0.05-1.13	0.07	0.56	0.11-2.93	0.49
≥ 60	0.86	0.12-6.44	0.89	1.01	0.22-4.66	0.99	1.17	0.26-5.37	0.84
Ethnicity									
Malay	1.00	—		1.00	—		1.00	—	
Chinese	2.88	0.50-16.68	0.24	2.97	1.09-8.14	0.03	3.32	1.32-8.33	0.01
Indian	1.72	0.30-9.77	0.54	1.89	0.52-6.93	0.33	2.70	0.71-10.30	0.15
Others	1.72	0.25-11.87	0.58	0.32	0.05-2.19	0.24	0.16	0.02-1.31	0.09
Residential area									
Urban	1.00	—		1.00	—		1.00	—	

Characteristic	Diabetes mellitus (n = 1365)			Hypertension (n = 2327)			Hypercholesterolemia (n = 1960)		
	aOR[1]	95% CI[1]	p-value	aOR[1]	95% CI[1]	p-value	aOR[1]	95% CI[1]	p-value
Rural	0.83	0.14-5.01	0.84	0.95	0.32-2.78	0.93	1.45	0.50-4.21	0.49
Marital status									
Single	1.00	—		1.00	—		1.00	—	
Married	0.24	0.02-3.40	0.29	0.48	0.08-2.71	0.40	0.40	0.08-2.06	0.28
Widow/Widower/Divorcee	0.23	0.01-7.44	0.40	0.26	0.03-2.50	0.24	0.29	0.03-2.48	0.26
Education level									
No formal education	1.00	—		1.00	—		1.00	—	
Primary education	0.59	0.07-4.76	0.62	0.77	0.10-6.07	0.81	0.45	0.06-3.63	0.46
Secondary education	0.70	0.05-9.66	0.79	1.15	0.13-10.00	0.90	0.77	0.09-6.31	0.81
Tertiary education	0.09	0.00-2.55	0.16	0.35	0.02-7.61	0.50	0.20	0.01-4.25	0.30
Occupation status									
Employed	1.00	—		1.00	—		1.00	—	
Unemployed	0.16	0.03-0.75	0.02	0.65	0.23-1.84	0.42	0.70	0.25-1.93	0.49
Monthly household income									
Bottom 40%	1.00	—		1.00	—		1.00	—	
Middle 40%	0.39	0.06-2.45	0.31	0.89	0.29-2.70	0.84	0.49	0.14-1.68	0.26
Top 20%	3.15	0.75-13.17	0.12	2.00	0.54-7.44	0.30	1.99	0.35-11.39	0.44
Smoking									

Characteristic	Diabetes mellitus (n = 1365)			Hypertension (n = 2327)			Hypercholesterolemia (n = 1960)		
	aOR[1]	95% CI[1]	p-value	aOR[1]	95% CI[1]	p-value	aOR[1]	95% CI[1]	p-value
Ever smoker	1.00	—		1.00	—		1.00	—	
Never smoker	0.55	0.12-2.46	0.43	0.83	0.25-2.69	0.75	0.61	0.20-1.88	0.39
Hypertension									
No	1.00	—		1.00	—		1.00	—	
Yes	12.66	1.06-150.64	0.05		NA		1.45	0.50-4.21	0.49
Hypercholesterolemia									
No	1.00	—		1.00	—		1.00	—	
Yes	0.99	0.25-3.91	0.99	1.35	0.46-3.96	0.59		NA	
Diabetes mellitus									
No		NA		—	—		—	—	
Yes				1.32	0.53-3.33	0.55	0.85	0.39-1.83	0.67

[1]aOR: adjusted odds ratio, 95% CI: 95% confidence interval. The model predicted value for diabetes mellitus, hypertension and hypercholesterolemia were 97.7% (area under curve (AUC): 0.65, 95% CI : 0.52–0.78, p = 0.03), 97.4% (area under curve (AUC): 0.68, 95% CI : 0.59–0.76, p = 0.04), and 97.1% (area under curve (AUC): 0.58, 95% CI : 0.48–0.68, p = 0.11)
